# High density linkage maps, genetic architecture, and genomic prediction of growth and wood properties in *Pinus radiata*

**DOI:** 10.1186/s12864-022-08950-6

**Published:** 2022-10-28

**Authors:** Jules S. Freeman, Gancho T. Slavov, Jakob B. Butler, Tancred Frickey, Natalie J. Graham, Jaroslav Klápště, John Lee, Emily J. Telfer, Phillip Wilcox, Heidi S. Dungey

**Affiliations:** 1 School of Natural Sciences, and ARC Training Centre for Forest Value, University of Tasmania, Private Bag 55, Hobart, TAS 7001 Australia; 2 Quantitative Genomics, Scion, Private Bag 3020, Rotorua, 3046 New Zealand; 3 Radiata Pine Breeding Company, Po Box 6213, Whakarewarewa, Rotorua, 3043 New Zealand; 4 School of Natural Sciences, University of Tasmania, Private Bag 55, Hobart, TAS 7001 Australia; 5 Bioinformatics, Scion, Private Bag 3020, Rotorua, 3046 New Zealand; 6 Present address: Te Uru Rākau -New Zealand Forest Service, Ministry for Primary Industries, Po Box 1340, Rotorua, 3040 New Zealand; 7 Department of Mathematics and Statistics, University of Otago, Dunedin, 9016 New Zealand

**Keywords:** Chromosomal rearrangement, Quantitative trait loci, Radiata pine, Single-nucleotide polymorphisms, Within-family genomic prediction

## Abstract

**Background:**

The growing availability of genomic resources in radiata pine paves the way for significant advances in fundamental and applied genomic research. We constructed robust high-density linkage maps based on exome-capture genotyping in two F_1_ populations, and used these populations to perform quantitative trait locus (QTL) scans, genomic prediction and quantitative analyses of genetic architecture for key traits targeted by tree improvement programmes.

**Results:**

Our mapping approach used probabilistic error correction of the marker data, followed by an iterative approach based on stringent parameters. This approach proved highly effective in producing high-density maps with robust marker orders and realistic map lengths (1285–4674 markers per map, with sizes ranging from c. 1643–2292 cM, and mean marker intervals of 0.7–2.1 cM). Colinearity was high between parental linkage maps, although there was evidence for a large chromosomal rearrangement (affecting ~ 90 cM) in one of the parental maps. In total, 28 QTL were detected for growth (stem diameter) and wood properties (wood density and fibre properties measured by Silviscan) in the QTL discovery population, with 1–3 QTL of small to moderate effect size detected per trait in each parental map. Four of these QTL were validated in a second, unrelated F_1_ population. Results from genomic prediction and analyses of genetic architecture were consistent with those from QTL scans, with wood properties generally having moderate to high genomic heritabilities and predictive abilities, as well as somewhat less complex genetic architectures, compared to growth traits.

**Conclusions:**

Despite the economic importance of radiata pine as a plantation forest tree, robust high-density linkage maps constructed from reproducible, sequence-anchored markers have not been published to date. The maps produced in this study will be a valuable resource for several applications, including the selection of marker panels for genomic prediction and anchoring a recently completed de novo whole genome assembly. We also provide the first map-based evidence for a large genomic rearrangement in radiata pine. Finally, results from our QTL scans, genomic prediction, and genetic architecture analyses are informative about the genomic basis of variation in important phenotypic traits.

**Supplementary Information:**

The online version contains supplementary material available at 10.1186/s12864-022-08950-6.

## Background

Recent advancements in genomic technologies, such as the increasing availability of genome sequencing and high-throughput molecular markers, coupled with falling costs, has seen the application of genomics rapidly gain in speed, magnitude and scope [1]. These advancements provide the opportunity for breakthroughs in fundamental and applied genomic research outside of model species, including in forest trees such as conifers [2, 3]. Dense genetic linkage maps constructed from sequence-anchored markers are invaluable for a variety of genomic applications, particularly in taxa lacking a complete genome assembly, as they provide important information regarding genome structure and recombination, which complements the finer scale information from genome-assemblies or genome-wide single-nucleotide polymorphisms (SNPs) [4]. For example, linkage maps in conifers have been successfully applied to comparative genomics [5], quantitative trait locus (QTL) analysis, candidate gene discovery and validation [6, 7]; and de novo whole genome assembly [4].

This study focuses on radiata pine (*Pinus radiata* D.Don), an important plantation species in temperate maritime regions of the southern hemisphere. *Pinus radiata* is native to the west coast of California (USA) and the Cedros and Guadalupe islands (Mexico), but has been widely planted elsewhere, including Chile, Australia and New Zealand [8]. In New Zealand, it occupies c. 90% of the 1.7 million hectares of planted forests (as of April 2019; MPI), and the Radiata Pine Breeding Company (RPBC) is currently prioritising genetic gain for volume, form, wood density, and stiffness [9].

With accelerating environmental change and economic uncertainty, there are strong incentives to accelerate the delivery of genetic gain and increase the agility of *P. radiata* breeding. This has driven significant investment in developing genomic resources, including an exome capture genotyping-by-sequencing panel [10], a genome assembly project [11], marker-based pedigree reconstruction, and genomic selection projects [12, 13]. However, robust high-density linkage maps have not yet been published for *P. radiata*. Better understanding of broad scale genome structure, linkage and ultimately haplotype structure in more diverse populations will be beneficial for many genomic applications. Such information will, for example, help refine the selection of markers for genomic applications and potentially improve the accuracy of genomic predictions [14]. Furthermore, the identification of QTL in biparental families is a first step toward a better fundamental understanding of trait genetic architectures.

The primary aim of this study was to produce robust high-density linkage maps for *P. radiata*. These linkage maps, along with the mapping populations and genotype and phenotype data generated for this study, were then used to dissect the genetic architecture underlying variation in growth and wood properties in *P. radiata*, including key traits relating to wood density and stiffness, and perform genomic prediction for growth and wood density. This research provides the foundation for a more detailed understanding of genome organisation, broad-scale recombination, and the genetic architecture of key traits targeted by tree improvement programmes.

## Methods

### Genetic material

Two full-sib F_1_ populations were used for map construction. Both have been described previously and are known as the ‘QTL’ (268405 × 268345, *n* = 94) and ‘framework’ (FWK; 850055 × 850096, *n* = 82) populations [10]. The parents of both populations were unrelated first-generation selections from unimproved New Zealand ‘landrace’ stands, which have been used to provide seed for commercial plantations. Genotype 268345 was also chosen for whole-genome assembly [11]. Both mapping populations were grown in the Bay of Plenty region of the North Island of New Zealand. The QTL mapping population was sampled from a large full-sib family planted in 1994 in a commercial plantation in Kinleith Forest. A subset of this family (1379 trees), from row plots in a single large contiguous block with similar aspect, was selected for phenotyping juvenile wood density (JWD) at age four. The 94 individuals used in this study were the extremes of JWD from this larger sample [15]. The FWK population was planted in 1978 in a genetic gain trial (compartment 1210, experiment RO1664/2) in Kaingaroa forest [16]. The trees used in this study were planted in a ‘pulpwood regime’ and were randomly sampled from six blocks of 64 trees (8 × 8), which were interspersed with other seedlots in a randomised complete block design. The FWK population has been used previously to construct moderate density linkage maps [17].

### DNA extraction and genotyping

DNA extraction and quantification were performed using the protocol developed by [18]. The germplasm used in this study represented a subset of the genotypes used in the development of the exome capture genotyping-by-sequencing panel for radiata pine; see [10] for details of DNA extraction, quantification and genotyping. Briefly, exome capture is a technique for sequencing the protein-coding regions of a genome, which provides a cost-effective alternative to whole-genome sequencing. It involves enrichment to selectively capture the exome using a panel of custom oligonucleotide probes, followed by sequencing with high throughput techniques. In this case a method developed in *Pinus taeda* and successfully used in other large conifer genomes was implemented [19], with probe development based on a large set of *P. radiata* transcriptome contigs [20] and the *P. taeda* reference genome [21].

### Filtering and ranking markers

After preliminary filtering described in [10], genotype data comprised 84,671 SNPs (genotyped in 95 progeny), and 75,413 SNPs (genotyped in 93 progeny), for the QTL and FWK mapping populations, respectively. Due to a high proportion of missing data, one of the QTL population progeny and 11 of the FWK population progeny were removed prior to filtering markers. Parental genotypes for each SNP were determined based on the majority call from 5 to 8 replicates. Following linkage map construction in the QTL population, this step was redone after it was discovered that some of the putative parental replicates for 268,345 were not of the expected genotype (see below).

These data were further filtered by removing markers: 1) in which both parents did not have a ‘reliable’ genotype call across the replicates (Additional file 1: Table S1), and 2) with > 20 missing data calls in the offspring. The remaining markers were divided into data sets segregating from each parent, and those segregating in a test-cross configuration (i.e., heterozygous in one parent and homozygous in the other) were retained for mapping (Additional file 1: Table S1). Only test-cross markers were selected for mapping as they have the greatest information content and produce more reliable marker orders than maps incorporating inter-cross markers [22]. Following this filtering, markers were ranked into different quality classes (Class 1–3) for mapping (see ‘Linkage map construction’ below), based on parental reproducibility, polymorphism information content (PIC; a measure of segregation ratio [23];) and missing data (Additional file 2: Table S2).

### Error correction and removal of redundant markers

#### Correcting genotyping errors

For each parental dataset, the genotype data were first re-coded into the double haploid data type required by SimpleMap [24]. The data were then loaded into Joinmap 4.1 [25] to estimate preliminary maps, with linkage groups defined at a minimum logarithm of the odds (LOD) score of 4 in each parental map. Ordering within linkage groups was performed using the maximum-likelihood algorithm with default parameters. The SMOOTH procedure [26] was then used to remove highly improbable data points, i.e. ‘singletons’ which are apparent double recombinants identified from neighbouring loci in the preliminary maps, which likely have a high proportion of errors. This procedure was repeated 16 times with increasingly strict error correction, following the method outlined in Van Os et al. [26]. In the first round data points were removed at a high singleton probability threshold of 0.99 then 0.98 and after this reducing by 0.02 each iteration until reaching a singleton probability threshold 0.70 at which point empirical evidence suggests the proportion of errors removed will be equal to the proportion of correct data points removed in error [26]. This resulted in c. 5–9% of genotype calls being re-coded as missing data within each parental dataset.

#### Binning markers

**Fig. 1:**
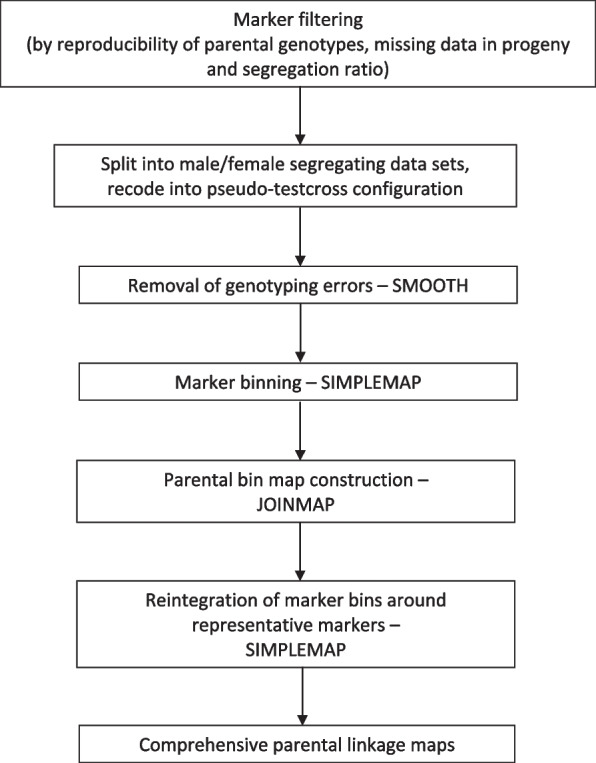
Summary of the pipeline used for the construction of parental linkage maps in the *Pinus radiata* QTL and FWK populations

After error correction, SimpleMap [24] was used to identify and remove redundant markers to increase the computational efficiency and improve the accuracy of linkage map construction. SimpleMap identifies bins of co-segregating or tightly linked markers. A maximum recombination threshold of < 3 cM Kosambi (roughly equivalent to 3% recombination frequency) is recommended for identifying ‘bins’ of linked markers in SimpleMap. Accordingly, bins were identified using a maximum of three recombinants in the QTL population, and two recombinants in the smaller FWK population. The SimpleMap pipeline identifies a single representative marker for each bin, which is placed in a file for mapping along with singletons (i.e., markers which did not bin). An additional file is also generated, which shows the markers within each bin and the number of recombinants between each marker and the representative marker, allowing reintegration and ordering of the removed markers around the representative marker after construction of bin maps (Fig. 1).

### Linkage map construction

Separate ‘bin maps’ were constructed for each parent using Joinmap 4.1, based on the representative markers for each bin as well as singletons which did not bin. Linkage groups were defined at a minimum of LOD 3. Marker ordering within groups was performed with the regression algorithm using default Joinmap 4.1 settings. An iterative procedure was used to construct linkage maps for each group, beginning with a subset of (c. 20–30) Class 1 markers, then adding smaller batches (ca. 1–6 markers) from the remaining Class 1 markers, before adding the Class 2 markers, and then Class 3 markers. The rationale behind this approach was to establish a ‘trusted order’ based on the highest quality markers before adding lower quality markers, which are likely to produce less reliable orders [27]. Joinmap produces up to three different maps at each attempt of marker ordering, with decreased stringency in round two and round three maps [25]. Following each iteration of mapping, the round one or, where present, round two maps were inspected and markers were removed based on whether they significantly changed the trusted order. Markers were also excluded based on: Chi-square goodness-of-fit contribution > 2.5; high nearest neighbour fit; the genotype probability function; being forced in a third round of mapping; and aberrant segregation distortion relative to surrounding markers, as all of these criteria can be indicative of genotyping errors. After removing markers according to these criteria, the maps were re-calculated and the above criteria re-evaluated until acceptable values were reached and all markers mapped within two rounds of mapping. Following the construction of bin maps, ‘comprehensive maps’ were constructed by reintegrating markers from the binning procedure around the representative bin markers using SimpleMap.

Linkage groups were numbered following a *P. taeda* reference linkage map [28], based on microsatellite (SSR) markers mapped in both *P. radiata* and *P. taeda*. In order to identify homologous linkage groups in our maps, SSR markers genotyped for an earlier comparative mapping study (unpublished), which included our QTL pedigree, were placed in the parental maps for 268405 and 268345. The SSR markers which were representative markers were all excluded due to the criteria described above, but a small number of bin markers remained in the final comprehensive maps of this pedigree. Shared exome-capture SNP markers at the contig level were used to standardise linkage group numbering and orientation in the FWK population c.f. the QTL population.

#### Refinements post-mapping

After completing the parental linkage maps we found that five of the 10 putative parental replicates used to determine the genotype for parent 268345 were incorrect, with samples originating from two different genotypes, one of which had been mis-labelled. Hence, the parental genotype at 268345 was retrospectively revised for all markers segregating in the QTL pedigree (from 268345 and 268405) based on the true replicates. Markers were removed from the linkage maps where the original genotype call changed or was not supported by a majority call from at least two replicates. For markers within bins, this involved simply removing the marker from the bin which did not affect the bin maps. Where the removed markers were representative markers, the maps were re-calculated and, where necessary, additional markers were excluded from the maps according to the above criteria (i.e., based on whether they significantly changed the trusted order and the other criteria for linkage map refinement).

#### Comparison of parental comprehensive maps

The repeatability of marker ordering was evaluated by calculating Spearman’s correlations between common syntenic markers (i.e., those that mapped to homologous linkage groups in each parental map) in all pair-wise combinations amongst the different parental linkage maps. ‘Common markers’ were identified at the contig level and where multiple markers from the same contig were mapped within a parental linkage group, the mean position of the markers representing a contig were used.

### Phenotypic measurements of the QTL population

#### Diameter at breast height and juvenile wood density

In the QTL population diameter at breast height (1.4 m; DBH [cm]) was measured at 5 years of age. Juvenile wood density (JWD [kg/m^3^]) was determined from two 5 mm diameter cores extracted 60 cm above ground level at 4 years of age. In cases where cores had more than trace amounts of compression wood (red colouration), they were discarded, and new cores extracted. Wood density in each core (JWD_A [kg/m^3^] and JWD_B [kg/m^3^]; Table 1) was estimated using the maximum moisture content method [29]. Where differences between the two cores exceeded 30 kg/m^3^, both cores were remeasured. Trees where differences between cores remained greater than 30 kg/m^3^ after remeasurement were removed from the experiment. QTL analysis was conducted on each core separately (i.e. JWD_A and JWD_B), as well as based on their arithmetic mean (JWD; Table 1).

**Table 1:** Phenotypic traits used for QTL analysis/validation and analyses of genetic architecture in each population of *Pinus radiata*

Trait	Description	Population	Genomic heritability (SD)	Predictive ability (SD)
^a^Silviscan traits				
Area	Ring area (mm^2^)	QTL	0.27 (0.17)	0.06 (0.10)
WD	Density (kg/m^3^)	QTL	0.71 (0.13)	0.57 (0.05)
Rad	Radial cell diameter (μm)	QTL	0.58 (0.18)	0.42 (0.06)
Tan	Tangential cell diameter (μm)	QTL	0.72 (0.12)	0.57 (0.04)
Crs	Fibre coarseness (μm/m)	QTL	0.31 (0.18)	0.10 (0.08)
Wall	Cell wall thickness (μm)	QTL	0.60 (0.16)	0.41 (0.05)
Sur	Specific surface area (m^2^/kg)	QTL	0.63 (0.16)	0.44 (0.05)
MFA	Microfibril angle (degrees)	QTL	0.48 (0.19)	0.29 (0.08)
MoE	Modulus of elasticity (GPa)	QTL	0.47 (0.19)	0.25 (0.08)
Other traits				
JWD_A	Density prediction for first 5 mm core (maximum moisture content method) (kg/m^3^)	QTL	0.71 (0.11)	0.55 (0.04)
JWD_B	Density prediction for second 5 mm core (maximum moisture content method) (kg/m^3^)	QTL	0.73 (0.11)	0.61 (0.04)
JWD^b^	Average of density predictions for two cores above	QTL	0.73 (0.10)	0.61 (0.04)
%LW1–10	Area weighted percent late wood ages 1–10 (%)	FWK		
WD^c^	Wood density (kg/m^3^)	Both	0.77 (0.08)	0.52 (0.04)
DBH^d^	Diameter at breast height (mm)	Both	0.19 (0.12)	0.11 (0.07)

^a^Assessed at 10 years of age

^b^Assessed at four years of age [15]

^c^Based on Silviscan ‘Den’ estimate in the QTL population and maximum moisture content estimate at 16 years of age in the FWK population

^d^Assessed at five years of age in the QTL population [15] and 19 years of age in the FWK population

#### Physical wood properties measured by Silviscan®

A range of physical wood properties were measured using the Silviscan®-2 system at the CSIRO Forestry and Forest Products laboratory (Melbourne, Australia). These measurements were based on a single 12 mm pith-to-bark core extracted 1.4 m above ground from each tree, at 10 years of age. Cores were soaked in ethanol prior to preparation of strips for measurement. Strips were prepared by cutting a 1 mm thick by 7 mm high flitch vertically, with respect to the grain, along the length of the core. Gravimetric density (weight/volume) was determined for each flitch. The flitches were then air dried (20 °C, 40% RH) and scanned for density using direct X-ray detection, and for microfibril angle (MFA [degrees]) using X-ray diffractometry. Density was obtained at 25 μm and 50 μm intervals and MFA was obtained at 5 mm intervals. All values were averaged within rings to obtain ring means. The modulus of elasticity (MoE [GPa]) was predicted for each core. Ring boundaries were allocated, using the wood density profile, and applied to the profiles of all other traits. The average properties for each growth ring were recorded for further analysis.

All data from the first measured ring closest to the pith were eliminated from further analyses due to minimum values of zero being detected for all traits in this ring. Following this, the data were weighted for ring area using the standard formula for calculating the area of a circle (area = *пr*^2^) and ring width data (r = ½ ring width + distance from pith to ring). Ring area weighting was undertaken because it reflects the true proportion of wood for each ring within the entire circumference of the tree at that height. Hence, area weighted means for each trait were used for QTL analyses, although correlations between area weighted and arithmetic means were very high (data not shown). Means were calculated for the area-weighted ring groups up to and including age 5, and for ring groups corresponding to 6–10 years of age. This resulted in no data for rings corresponding to ages 1 and 2, sometimes for age 3, and occasionally age 4. This was because there were no data for the inner ring, due to the trees not reaching a height of 1.4 m (breast height) by their first, and sometimes second, year. Also, data for the inner-most ring on the cores were eliminated from the analyses because of zero values from SilviScan®, as described above.

The following SilviScan® traits were used for QTL analyses: (Table 3): ring area (Area [mm^2^]); wood density (WD [kg/m^3^]); radial (Rad [μm]) and tangential (Tan [μm]) cell diameters; fibre coarseness (Crs [μm/m]); cell wall thickness (Wall [μm]); specific surface area (Sur [m^2^/kg]); MoE [GPa]; and MFA [degrees] (Table 1).

### Phenotypic measurements in the FWK population and across populations

In the FWK population, DBH and WD were measured at 16 years of age, while the percent of late wood for ages 1–10 (%LW1-10) was measured from cores extracted at 19 years of age. To measure WD, two 5 mm outer wood cores of 50 mm length were taken from opposite sides of each tree, and density was measured using the maximum moisture content method described above. Least square means for WD were used for QTL analyses to account for variation between replicate blocks, and random error, within the field trial [16]. To measure %LW1-10, two 5 mm diameter cores were extracted 1.4 m above ground level at 19 years of age. Traits related to wood density, including %LW1–10, were estimated using x-ray densitometry [30]. The percentage of latewood in rings corresponding to years 1–10 was weighted for ring area as described above. Descriptive statistics and frequency distributions for all phenotypic traits measured in each population are shown in Additional file 3: Table S3 and Additional file 4: Fig. S1, respectively.

For genomic prediction and analyses of genetic architecture (see below), phenotypic values for DBH and WD across both populations were then calculated as best linear unbiased predictors (BLUPs) using a mixed linear model, which was implemented in the lme4 R package [31, 32] and included “Experiment” (effectively mapping population) as a fixed effect and “Genotype” as a random effect. In addition, we also performed analyses of Silviscan traits (as defined above) within the QTL population.

### QTL analysis

QTL discovery was performed in the QTL population for Silviscan traits, JWD (four years of age) and DBH. QTL analyses in the FWK population for stem diameter (DBH) and wood density traits (WD and %LW1–10) were used to validate the genomic location of QTL from the QTL population.

In both mapping populations, QTL analyses were conducted with MAPQTL 6.0 [33] based on the individual parental bin maps. Analyses used interval mapping (IM) with the regression algorithm and MAPQTL 6.0 [33] default parameters. In the QTL population (used for QTL ‘discovery’), map intervals exceeding the suggestive significance threshold in IM were selected as cofactors for restricted multiple-QTL model (rMQM) mapping. rMQM mapping was performed using an iterative approach and forward selection of cofactors until a stable set of cofactors was found and no further QTL were detected. In both populations and analyses, putative QTL were declared at two different levels: suggestive (chromosome-wide type I error < 0.05) and significant (genome-wide type I error < 0.05). The LOD thresholds for each level were determined based on 1000 permutations [34]. QTL were presented at the reduced ‘suggestive’ significance threshold to aid comparative mapping [35], as this significance level will decrease the type II error rate relative to the genome-wide significance threshold. The non-parametric Kruskal-Wallis test was used to provide additional support for the QTL found in the QTL population. This approach makes no assumptions about the probability distribution of the quantitative trait after fitting the QTL genotype [33]. All input files used for QTL analysis are included as Additional files (5, 6, 7, 8, 9, 10, 11, 12, 13 and 14).

#### QTL validation

For validation, the location of QTL from equivalent linkage groups in each population were compared based on the closest ‘common marker’ to each QTL peak (i.e., markers from the same contig included in the parental maps of both pedigrees; where multiple markers from the same contig mapped to the same parental linkage group, their mean position was used). In cases where the closest common marker differed between the QTL in each population, the mean distance between these markers in each parental map was estimated. To account for genomic regions with few markers, and localised inconsistencies in marker order between maps, threshold values of < 15 cM between the QTL peak markers and the closest common marker in each map, and < 5 cM mean distance between the closest common markers in each map, were used to declare QTL validation.

### Genomic prediction and analyses of genetic architecture

We used standard ridge regression/GBLUP genomic prediction methodology [36, 37] as implemented in the rrBLUP R package [38] and random cross-validations [39] to calculate the predictive ability (the correlation of predicted and “observed” phenotypes for the validation population) of GBLUP as applied to unknown phenotypes in the QTL and FWK populations. In contrast to a previous study Klápště et al. [40], in which we used a much wider training population, both training and test sets were randomly drawn (i.e., according to the desired training population size) only from the respective mapping population. This simulates a breeding scenario where a small subset of a full-sib family is field-tested to obtain phenotypic data, and then a family-specific GBLUP model is used to predict phenotypes for a larger subset of genotypes that were either stored (e.g., cryopreserved) or obtained by repeating the original controlled cross. In addition, we used the Bayesian Mixture Model “BayesR” [41] as implemented in the GCTB software [42] to estimate genomic heritabilities, genetic architecture parameters, and marker effect sizes. We ran BayesR using its default parameters in GCTB, partitioning markers into four classes: no effect, small, medium, and large effects [*γ* = (0,0.01,0.1,1.0)’], where *γ* are the scaling factors for the respective marker effect variances ${\sigma}_{\beta}^2$ of each class, with marker effects defined as the partial regression coefficients (*β*) of markers on phenotypes, expressed in phenotypic standard deviations. The prior for the mixing proportions of the four classes was the default *π* = (0.95,0.03,0.01,0.01).

## Results and discussion

### Linkage mapping

Of the testcross markers segregating from each parent, we were able to place in the comprehensive parental linkage maps 1285 and 4487 (27 and 41%) for 268345 and 268405, respectively; and 4674 and 4132 (50 and 41%) for 850055 and 850096, respectively (Table 2, Additional file 15: Table S4). All maps had 12 linkage groups, corresponding to the haploid chromosome number in *P. radiata*. Marker density was generally high, with the mean interval between markers ranging from c. 0.7–1.0 cM among all parental maps except 268345, in which the mean interval was 2.1 cM. The lower number of markers and marker density in the 268345 parental map reflected the apparent lower number of markers segregating from this individual. However, the apparent low segregation from parent 268345 was due to errors among the putative parental replicates used for the initial filtering in this parent, which led to many markers being excluded in the initial filtering and to a much lesser degree retrospectively (see ‘Refinements post mapping’ above). Specifically, after the initial filtering, there were 4844 test-cross markers which were used for mapping in 268,345 (results not shown), although the second filtering post mapping showed there should have been 11,599 markers available for mapping from this parent (Additional file 1: Table S1). Overall, there was a low proportion of redundancy [based on the assignment of exome capture markers to *P. taeda* contigs [10];] among the parental maps with a total of 7123 unique *P. taeda* contigs placed across the four comprehensive parental maps, which is promising for the effective anchoring of scaffolds in the *P. radiata* genome assembly (not available at the time of this analysis). The overall marker orders were generally stable between iterations of map construction, and rank order correlations among syntenic markers in the comprehensive parental maps were high (mean Spearman's *r* = 0.94–0.97; Table 3), giving confidence that marker orders were robust. Map lengths were similar to previous linkage maps constructed in *P. radiata* [17], and smaller than the recent ultra-dense maps constructed in *Picea abies* [4].

**Table 2:** Summary statistics for comprehensive maps in the *Pinus radiata* QTL and FWK pedigrees^a^

Population	Parent	# Markers	Unique positions	Linkage group length (cM)	Total length (cM)	Mean interval between markers (cM)
**QTL**	268,345	1285	1095	147.6–237.1	2291.9	2.11
**QTL**	268,405	4487	3173	142.2–199.4	2117.9	0.67
**FWK**	850,055	4674	2355	84.0–174.3	1642.7	0.70
**FWK**	850,096	4132	2019	121.0–245.1	2081.7	1.04

^a^cM values are cM Kosambi

**Table 3:** Spearman’s correlations of marker order between *Pinus radiata* parental linkage maps^a^

LG^**b**^	850,055 vs 850,096	850,055 vs 268,345	850,055 vs 268,405	850,096 vs 268,345	850,096 vs 268,405	268,345 vs 268,405
**1**	0.94 (39)***	0.93 (25)***	0.97 (58)***	0.93 (23)***	0.96 (59)***	0.97 (23)***
**2**	0.97 (65)***	0.99 (41)***	0.97 (75)***	0.99 (21)***	0.96 (60)***	0.95 (17)***
**3**	0.97 (37)***	0.95 (19)***	0.96 (50)***	0.97 (11)***	0.94 (43)***	0.86 (11)**
**4**	0.94 (19)***	0.97 (23)***	0.99 (44)***	0.99 (10)***	0.95 (25)***	0.90 (15)***
**5**	0.98 (29)***	0.96 (18)***	0.98 (50)***	0.98 (17)***	0.93 (42)***	0.95 (10)***
**6**	0.90 (9)***	1.00 (8)***	0.97 (37)***	0.95 (8)**	0.96 (31)***	0.89 (7)*
**7**	0.94 (23)***	0.90 (5)^n.s.^	0.97 (51)***	0.93 (7)**	0.95 (42)***	1.00 (6)**
**8**	0.81 (32)***	0.93 (12)***	0.98 (54)***	0.95 (8)**	0.95 (36)***	1.00 (4) ^n.s.^
**9**	0.95 (51)***	0.97 (13)***	0.97 (43)***	0.95 (20)***	0.98 (50)***	0.80 (4) ^n.s.^
**10**	0.88 (17)***	0.84 (10)**	0.91 (38)***	0.92 (11)***	0.97 (49)***	0.98 (14)***
**11**	0.97 (27)***	0.99 (16)***	0.98 (42)***	0.89 (12)***	0.98 (39)***	1.00 (7)***
**12**	0.97 (30)***	0.95 (18)***	0.98 (54)***	1.0 (7)***	0.95 (41)***	1.00 (6)**
**Mean**	0.94 (31.5)	0.95 (17.3)	0.97 (49.7)	0.95 (12.9)	0.96 (43.1)	0.94 (10.3)

^a^Based on *P. taeda* contigs common to parental maps. Where multiple markers represented a single contig, the mean position of these markers was used. (#) = The number of contigs used in the comparison for each linkage group. * = *P* < 0.05, ** = *P* < 0.01, *** = *P* < 0.001

The large number of molecular markers produced by high-throughput technologies challenges conventional approaches for linkage mapping, both in terms of computing time and the accuracy of ordering tightly linked markers. Even relatively small amounts of genotyping errors impact marker ordering [43] and greatly inflate map lengths in high-density maps [44]. The impact on marker ordering can extend beyond tightly linked markers to cause inversions [43] and translocations of map regions > 10 cM. Our method of first correcting improbable genotypes, then removing redundant (and near redundant) data from map calculations proved highly effective in achieving robust marker orders, as evidenced by high repeatability during mapping iterations, high rank order correlations among the comprehensive parental maps, and realistic map lengths. Correcting genotyping errors also significantly reduced overall map lengths in our study, which were at least an order of magnitude greater prior to error correction compared to the final maps and expectations based on previous linkage maps in *P. radiata* [7, 17].

### Synteny amongst parental linkage maps

Comparison of common markers between parental maps (at the contig level) revealed a significant departure from synteny among the parental linkage maps (Additional file 16: Fig. S2). Specifically, ‘linkage group 8’ in parent 850,096 was ‘chimeric’, being composed of markers which mapped to both linkage groups 8 and 10 in the 268345 and 268405 parental linkage maps. The distribution of syntenic markers was quite striking, with 0 to ~ 155 cM on parent 850096 linkage group 8 composed of markers homologous to linkage group 8, and a further ~ 90 cM composed of markers homologous to linkage group 10 in parents 268345 and 268405, with high collinearity among the parental maps in each distinct region. Where these different regions intersected, in parent 850096 linkage group 8, there was a region of ~ 10 cM with a mixture of homologs from linkage groups 8 and 10 as well as very tightly clustered markers (Additional file 16: Fig. S2B). In contrast, all mapped markers in parent 850096 linkage groups 8 and 10 matched only their homologous chromosomes in the mapped markers from parent 850,055 (Additional file 16: Fig. S2C).

The departure from synteny in 850096 relative to the other parental linkage maps was somewhat surprising given the high level of synteny previously found between linkage maps in *P. radiata* [7] and more broadly between *Pinus* and *Picea* (family Pinaceae) species [45]. Reciprocal translocations are known to cause pseudo-linkage between markers on different chromosomes in linkage maps produced from the offspring of individuals heterozygous for the translocations [46–48], which may explain the chimeric linkage group 8 in 850096. Other potential explanations for the departure from synteny among the parental linkage maps include: i) mapping error; or ii) translocation of a region of linkage group 10 to linkage group 8 in parents 268345 and 268405. To investigate the possibility that the departure from synteny was an artefact of mapping error, we checked statistical support for defining linkage groups and ordering markers within linkage groups 8 and 10 in 850096. However, there was no evidence for mapping error as both linkage groups: i) remained stable to a high independence LOD (minimum of LOD 8) in the grouping tree; and ii) had strong statistical support for marker order from criteria such as the Chi-square goodness-of-fit contribution, and the genotype probability function. Furthermore, inspection of the initial marker grouping in parent 850055 linkage group 10 (as opposed to the final map for this group) revealed numerous markers representing contigs which mapped to linkage group 8 and 10 in parent 850096 but not the other parents, supporting the presence of a chimeric linkage group in parent 850096 and arguing against an inter-chromosomal translocation in 268345 and 268405. Therefore, of the above potential explanations the most parsimonious one for the chimeric linkage group in 850096, and the tightly clustered markers where homologs from both of these groups are found, is that this parent is heterozygous for a reciprocal translocation involving linkage groups 8 and 10. Notably, inspection of the initial grouping for linkage group 10 in parent 850055 also showed markers covered a larger area of linkage group 10 than those in the final maps (Additional file 16: Fig. S2A, Fig. S2C). Hence, in this case ‘mapping error’, i.e., the exclusion of many poorly fitting markers during map construction (see linkage map construction above), very likely explains the low coverage of linkage group 10 in parent 850055.

Chromosomal structural rearrangements are common throughout the evolutionary history of land plants, including conifers [5, 45, 49]. Reciprocal translocations are among the most common structural rearrangements and have important consequences for recombination, adaptation and gamete fertility [47]. They have been detected in many species by techniques such as: studying pollen viability; chromosome pairing during meiosis; linkage mapping; and DNA sequence analysis [46–48]. The occurrence of a large linkage group with markers homologous to multiple groups in a reference genome, or linkage maps of other individuals, as well as very tightly clustered markers indicative of suppression of recombination around the breakpoints, is consistent with observations in other species for which there are multiple lines of evidence for reciprocal translocations, including barley, soybean, and hybrids between peach and almond [46–48]. Further work will be required to provide additional evidence for the putative chromosomal rearrangement in 850,096 and its potential functional implications.

### QTL analysis

**Fig. 2:**
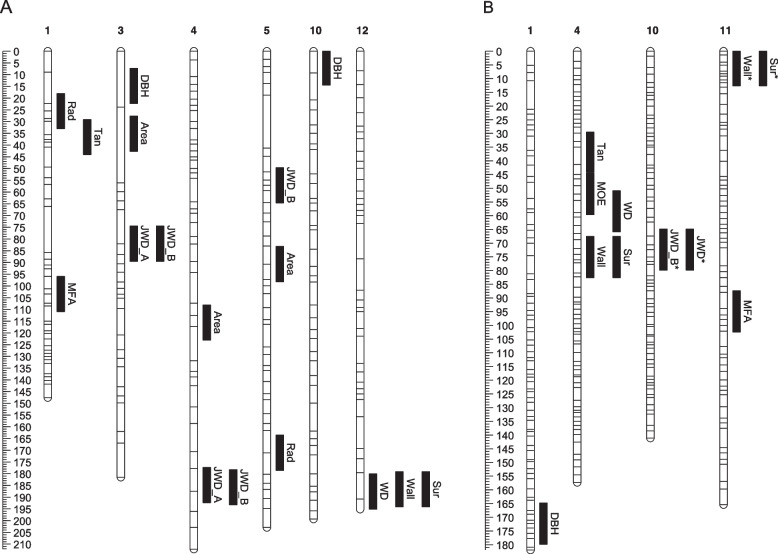
QTL positions on the parental linkage maps of the *Pinus radiata* QTL population. **A** QTL positions in the 268,345 parental linkage map. **B** QTL positions in the 268,405 parental linkage map. Scale bars shows cM (Kosambi). Horizontal lines show the location of markers in the parental bin maps

**Fig. 3:**
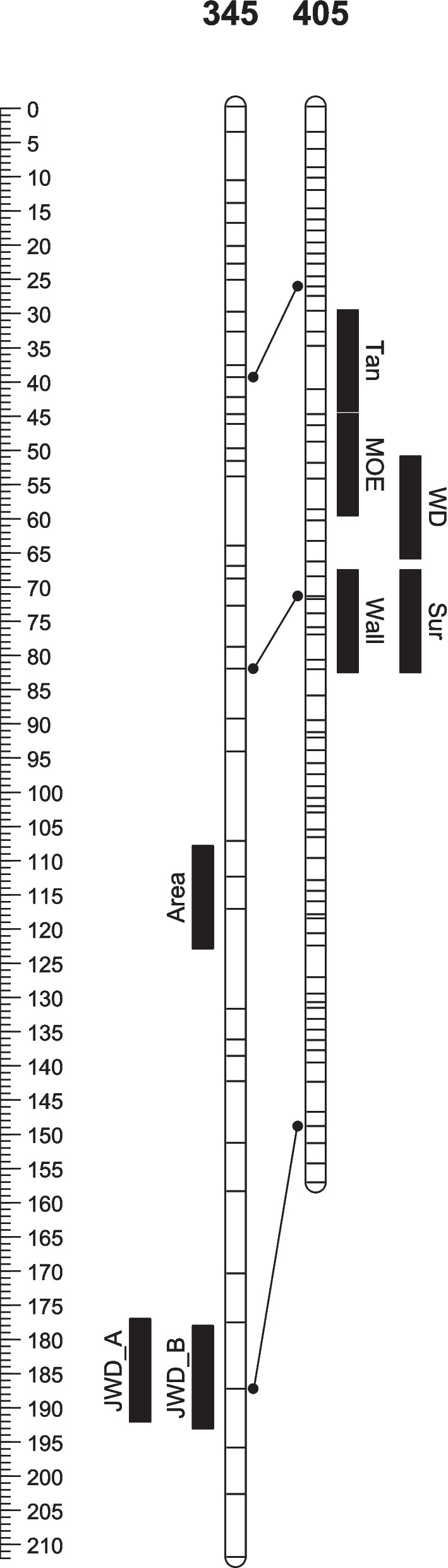
QTL positions on linkage group 4 in the 268345 and 268405 parental linkage maps in the *Pinus radiata* QTL population. Scale bar shows cM (Kosambi). Horizontal lines show the location of markers in the parental bin maps. Lines between groups show the location of a sub-set of homologous contigs between the parents

QTL ‘discovery’ was performed in the QTL population while the FWK population was used to provide positional ‘validation’ of the QTL discovered. In total, 28 QTL were detected in the QTL population, five of which were significant at the genome-wide level (type I error rate < 0.05). The remaining 23 were significant at the suggestive level only (chromosome-wide type I error rate < 0.05), however all but two of the 28 QTL were supported by the non-parametric Kruskal-Wallis tests (Table 4). Within each parent, 1–3 QTL were detected per trait, and the estimated phenotypic variation explained by each QTL ranged from 8.4–19.1% (Table 4). Despite some co-colocations of QTL for different traits within each parental map (defined as having peaks within 7.5 cM), QTL were widely distributed across 7 of the 12 linkage groups (Fig. 2). Specifically, 17 QTL mapped to 13 discrete regions in six linkage groups in the 268,345 parent, and 11 QTL mapped to 8 discrete regions on 4 linkage groups in the 268,405 parent. Not surprisingly, most of the co-located QTL were for traits with very high phenotypic correlations in the QTL population, such as Sur and Wall, and JWD measurements (Additional file 17: Table S5). Notably, comparison of homologous markers between linkage maps suggested no QTL were co-located between the different parental maps (Fig. 2, Fig. 3). However, four QTL in two different genomic locations (defined as regions with QTL separated by no more than 1 cM) in the discovery population, were also detected in the ‘validation’ FWK population at the suggestive significance level (Table 4). Both of these QTL segregated from the 268,405 parent (Table 4, Fig. 2B).

**Table 4:** QTL for growth and wood properties detected by rMQM mapping in the *Pinus radiata* QTL population

Trait	Population and parent	LG	cM^**a**^	Adjacent marker^**b**^	LOD^**c**^	PVE^**d**^	KW Support^**e**^	Validated^**f**^
**Silviscan traits**^**f**^								
**WD**	QTL/405	4	58.4	63204602_117107	3.44	16.7	****	
**Tan**	QTL/405	4	37.0	63055824_6340	2.56	12.7	**	
**Wall**	QTL/405	4	76.1	63177503_30587	4.40**	18.9	**	
	QTL/405	11	5.1	62510986_25015	2.80	11.5	*	DBH (4.85)
**Sur**	QTL/405	4	75.1	63177503_30587	4.45**	19.1	**	
	QTL/405	11	5.1	62510986_25015	2.82	11.6	*	DBH (4.85)
**MFA**	QTL/405	11	94.9	62955572_13326	2.82	13.9		
**MOE**	QTL/405	4	52.1	63204602_174680	2.32	11.5	*	
**Other traits**								
**JWD_B**	QTL/405	10	72.4	58974239_18736	2.43	12.0	***	%LW1-10 (0)
**JWD**	QTL/405	10	72.4	58974239_18736	2.37	11.8	**	%LW1-10 (0)
**DBH**	QTL/405	1	172.3	62736216_20622	2.73	13.7	**	
**Silviscan traits**								
**Area**	QTL/345	3	35.1	62962718_10700	3.09*	12.2	**	
	QTL/345	4	115.6	62596398_9713	2.55	9.9	*	
	QTL/345	5	90.7	63227690_179297	2.39	9.4	**	
**WD**	QTL/345	12	187.6	63206125_97028	2.23	11.2	*	
**Rad**	QTL/345	1	25.5	63090351_5536	2.82	12.2	**	
	QTL/345	5	171.1	62991642_9036	2.11	9.0	**	
**Tan**	QTL/345	1	36.6	63118470_4543	2.09	10.5	**	
**Wall**	QTL/345	12	186.6	63206125_97028	2.49	12.3	*	
**Sur**	QTL/345	12	186.6	63206125_97028	2.47	12.3	*	
**MFA**	QTL/345	1	103.4	63206856_147794	2.71	13.4	**	
**Other traits**								
**JWD_A**	QTL/345	4	184.8	63016409_79396	2.87	12.8	**	
	QTL/345	3	82.0	62923239_33293	2.46	11.0	*	
**JWD_B**	QTL/345	4	185.8	63016409_79396	3.38*	13.8	**	
	QTL/345	5	57.1	62362541_2029	2.52	10.1	*	
	QTL/345	3	82.0	62923239_33293	2.11	8.4		
**DBH**	QTL/345	3	14.8	62962718_10700	3.81*	17.4	**	
	QTL/345	10	7.0	63227371_228101	2.38	10.8	*	

^a^QTL LOD peak position

^b^Closest marker to QTL LOD peak

^c^Genome wide significance * = *P* < 0.05, ** = *P* < 0.01. The remaining QTL were significant at the suggestive level (chromosome-wide type I error rate < 0.05)

^d^The percent variation explained at each QTL peak

^e^Kruskal-Wallis significance at the closest marker (ie adjacent marker) to each QTL peak. * *P* < 0.05, ** *P* < 0.01, *** *P* < 0.001, **** *P* < 0.0001

^f^Positonal support for QTL from those found in the FWK population, all of which were significant at the suggestive level. In each case, the trait for which the supporting QTL was found is shown, (#) indicates average distance to the closest shared marker to each of the QTL in each map

^f^Silviscan traits are an average from two measurements: tree-rings 1–5 and tree-rings 6–10

The low number of QTL detected for each trait (mean 1.6/parent, where significant QTL were found), and the relatively high estimated range of phenotypic variance explained (PVE) by each QTL (> 10% for 24 of the 28 QTL discovered; Table 4) likely reflects the relatively small sample size of the QTL population, which is a common limitation in QTL studies of forest trees [50]. Specifically, the small sample size employed will reduce the number of QTL detected per trait, and overestimate the effect size of those QTL that are detected [51], both of which will bias the findings to suggest ‘major effect’ QTL are segregating in this pedigree. Indeed, Hall et al. [50] estimated the ‘effective number of QTL’ (based on meta-analysis of QTL studies in forest trees) to be 17 and 35, with a median PVE of individual QTL 4.5 and 7.7, for traits related to growth and physical wood properties, respectively, and that c. 1800 individuals are needed to capture 50% of the genetic variation in these traits. The selection of extreme phenotypes for wood density in the QTL population [15], however, likely increased the statistical power to detect QTL for wood density and correlated traits, such as cell wall thickness and specific surface area, relative to a random population of this size [52]. Nonetheless, for all traits studied there are no doubt many more loci influencing the phenotypic variation in the mapping population that were not detected, in line with previous findings particularly for ‘complex traits’ such as growth (DBH and or height) and wood density from QTL [15] and genome-wide association studies [53, 54]. Despite this, the QTL presented here, particularly in the two genomic regions with positional support from the FWK population, provide promising targets to search for candidate genes and for further functional genomic studies. We are currently conducting genome-wide association and QTL mapping studies employing much larger sample sizes and more diverse germplasm to better characterise the number, location and magnitude of effects of loci influencing growth and wood properties in the radiata pine breeding programme.

### Genetic architecture and genomic prediction

**Fig. 4:**
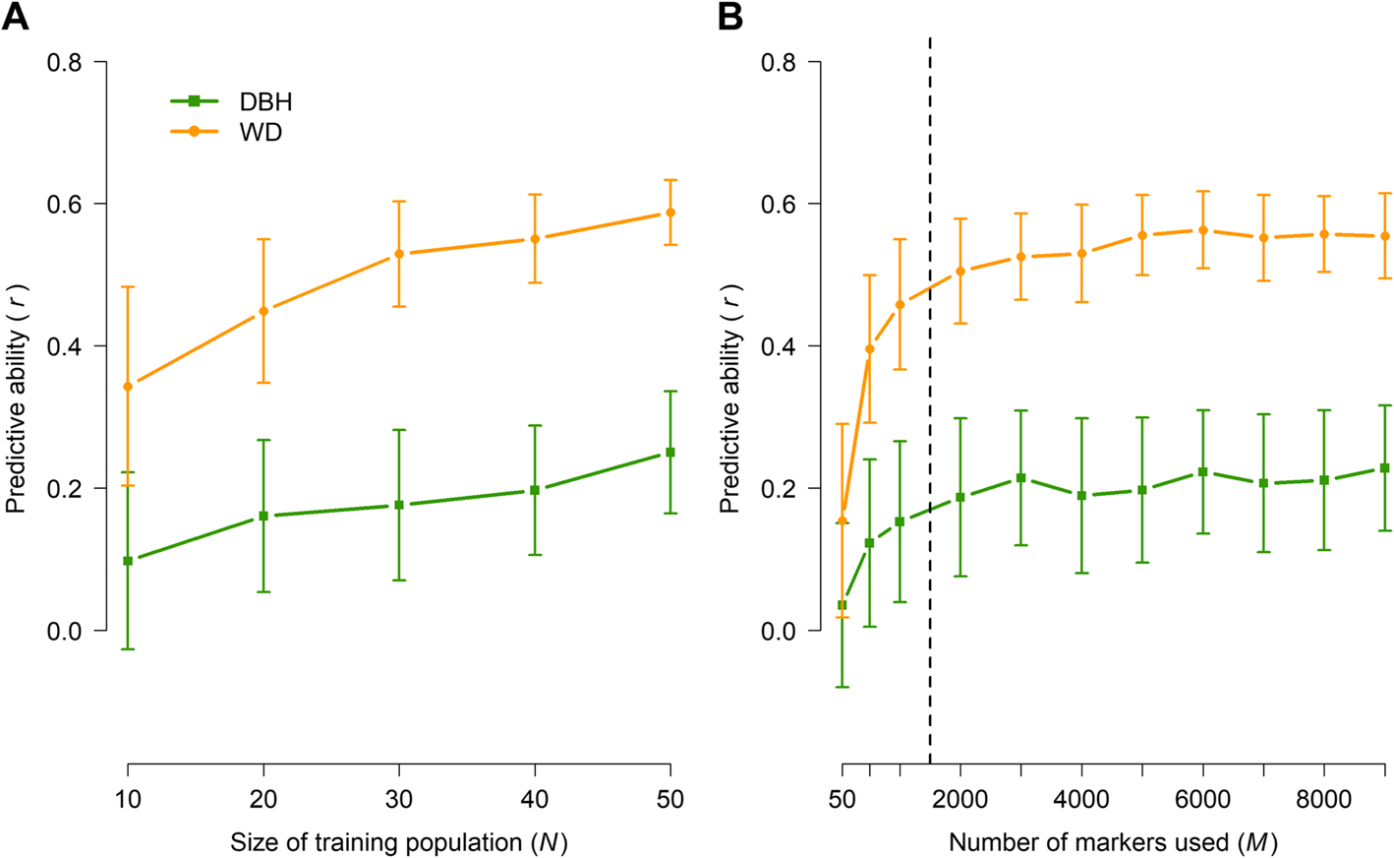
Genomic predictive ability for diameter at breast height (DBH) and wood density (WD) as a function of training population size (**A**) and number of markers (**B**) used in random cross-validations of 86 *Pinus radiata* genotypes from the QTL population. Error bars correspond to standard deviations across 100 random cross-validations for each set of parameters. Analyses in (**A**) were based on all markers (*M* = 9353). The training population size in (**B**) was *N* = 43 (two-fold cross-validation) and subsets of markers were selected at random

**Fig. 5:**
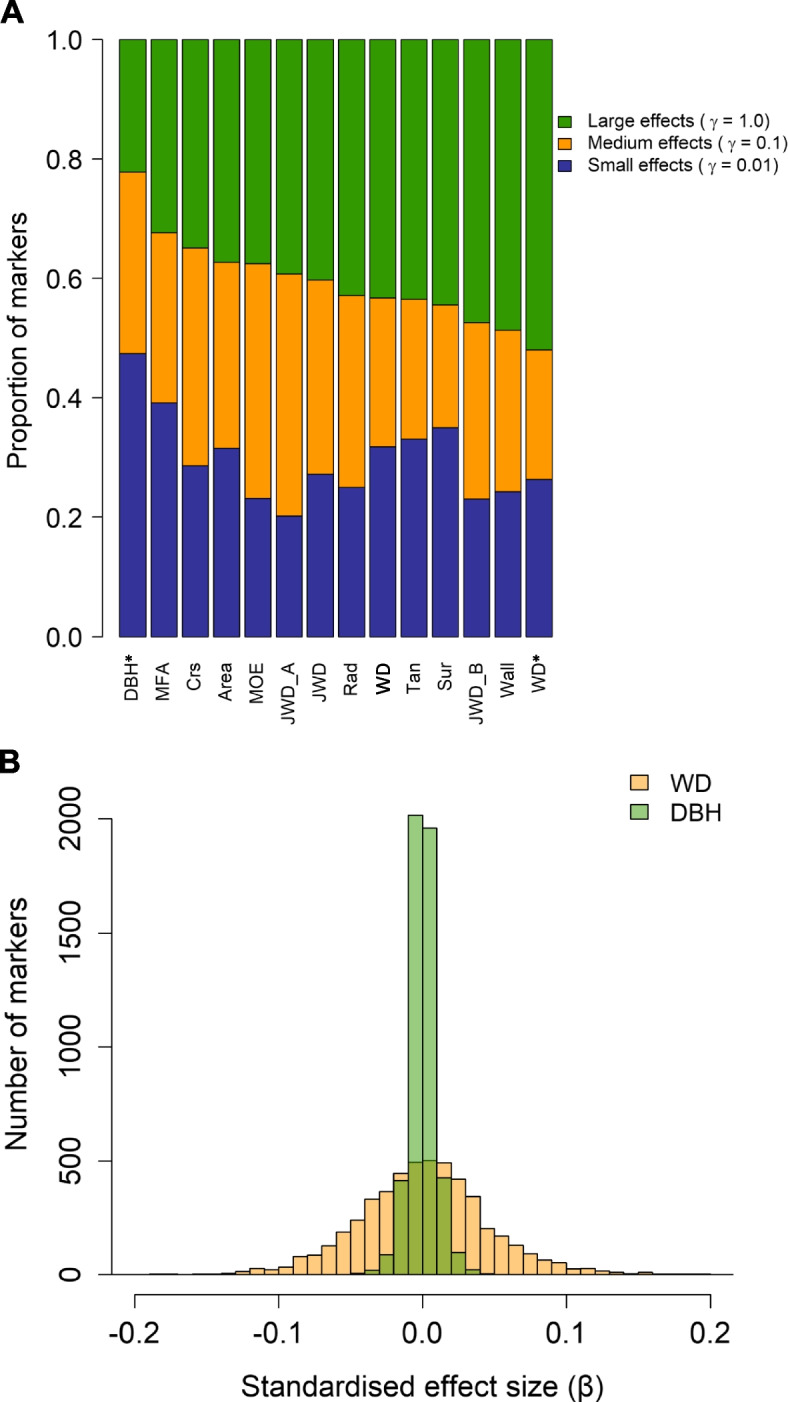
Genetic architecture of growth and wood quality traits in *Pinus radiata*. Relative proportions of markers with small, medium, and large effects (**A**) and standardised marker effect size distributions for diameter at breast height (DBH) and wood density (WD) as estimated using GCTB (**B**). Figure 5 (**B**) reports results from analyses across both populations. In Fig. 5 (**A**) WD* and DBH* were from analyses combined across populations, the remaining traits were from the QTL population only

Results from genomic prediction and analyses of genetic architecture (Table 1, Figs. 4 and 5, S2) were generally consistent with those from QTL scans. There was substantial variation among traits, with wood characteristics generally having moderate to high genomic heritabilities and predictive abilities (Table 1) and relatively less complex genetic architectures (i.e., controlled by higher proportions of loci with large and moderate effects, Fig. 5) compared to DBH. This is not surprising as numerous previous studies have shown that DBH, presumably a highly complex trait, with relatively weak genetic control and strong genotype-by-environment interaction in comparison to wood characteristics (reviewed by [55]) and is therefore a challenging trait to predict using genomic information [56–58]. Similarly, low predictive ability was observed for height in Norway spruce [58], in line with the detection of few QTL in later genome-wide association studies for this trait. The authors attributed the lack of power to detect QTL to the implemented genomic resources (exome capture might miss some important regulatory regions) and the size of the discovery population [59]. Overall, our results are consistent with a growing consensus that marker-assisted selection will likely have limited applicability in tree breeding because of the high complexity of most traits of interest, with genomic selection being the only potentially feasible approach at present [3].

Our findings on within-family genomic prediction complement results from a previous study, in which we used a much wider training population and the resulting within-family predictive abilities were moderate for WD, but very low or absent for DBH [40]. This was also the case when adding up to 40 individuals from the respective mapping family to the wider training population [40]. In contrast, the alternative approach we assessed here (i.e., training GBLUP models specifically within each family), resulted in predictive abilities that increased stably with training population size and reached a plateau with ca. 2000 markers for both WD and DBH (Fig. 4, Additional file 18: Fig. S3). Taken together, results from these two studies have two practical implications for the implementation of genomic selection. First, using family-specific GBLUP models for within-family prediction is clearly superior for more complex and less heritable traits, such as DBH. This is presumably because the genetic architectures of such traits, even if completely unknown at present, are likely to vary considerably among populations and families (i.e., because different sets of causative loci segregate in each family or population). Implementing family-specific GBLUP models will almost certainly be unfeasible in typically structured tree breeding programmes because of the logistical challenges associated with holding large numbers of seedlings or cryopreserved somatic embryogenesis materials until phenotypic data become available to train these models. However, knowing that reasonable predictive abilities can be achieved even with very small training population sizes (Fig. 4, Additional file 18: Fig. S3) would make it possible to revisit particularly successful controlled crosses retrospectively and perform within-family genomic selection with very high intensity (i.e., select the very best full-sibling from the very best control-pollinated families). Such scenarios could conceivably be exploited in the ‘rolling front’ radiata pine breeding programme in New Zealand [60], in which clonal tests of control-pollinated offspring produced by the top parents are established every year. Second, practically useful genomic predictive abilities were achievable using as few as 2000 SNPs both within the two families used in this study (Fig. 4, Additional file 18: Fig. S3) and across much wider breeding populations [40]. Thus, low-density genotyping platforms can considerably improve the cost-benefit ratio of ongoing genomic selection experiments in radiata pine.

## Conclusion

The linkage maps produced in this study provide fundamental information regarding linkage and genome structure in *P. radiata*, which will be useful for a variety of applications including selection of marker panels for genomic studies (such as genomic selection) and comparison with our de novo whole genome assembly. Comparison of common markers in the parental linkage maps provides the first map-based evidence for a large genomic rearrangement in *P. radiata*. The QTL presented here will be useful for further genomic studies, such as candidate gene discovery and validating association genetics findings. Finally, genomic prediction and genetic architecture analyses provide insights into the genomic basis of variation in important phenotypic traits and inform practical decisions regarding the implementation of genomic selection in radiata pine breeding.

## Supplementary Information

**Additional file 1: Table S1.** SNP filtering summary for the *Pinus radiata* QTL and FWK mapping populations.

**Additional file 2: Table S2.** SNP ranking criteria used for the *Pinus radiata* QTL and FWK mapping populations.

**Additional file 3: Table S3.** Descriptive statistics for the phenotypic traits measured in the QTL and FWK *Pinus radiata* populations in this study.

**Additional file 4: Fig. S1.** Frequency distributions for the phenotypic traits measured in the QTL and FWK *Pinus radiata* populations in this study. QTL population: (A) Ring area (mm^2^); (B) Density (kg/m^3^) Silviscan; (C) Radial cell diameter (μm); (D) Tangential cell diameter (μm); (E) Fibre coarseness (μm/m); (F) Cell wall thickness (μm); (G) Specific surface area (m^2^/kg); (H) Microfibril angle (degrees); (I) Modulus of elasticity (GPa); (J) Density prediction for first 5 mm core (maximum moisture content method) (kg/m^3^); (K) Density prediction for second 5 mm core (maximum moisture content method) (kg/m^3^); (L) Average of density predictions for two cores above; (M) Diameter at breast height (mm). FWK population: (N) Area weighted percent late wood ages 1–10 (%); (O) Wood density (kg/m^3^) (maximum moisture content method); (P) Diameter at breast height (mm).

**Additional file 5.** 268,345 map file. Plain text file containing the map position (cM) of all loci on the 268,345 linkage map, in the format required for analysis using MAPQTL 6.0.

**Additional file 6.** 268,405 map file. Plain text file containing the map position (cM) of all loci on the 268,405 linkage map, in the format required for analysis using MAPQTL 6.0.

**Additional file 7.** 268,345 loc file. Plain text file containing the genotype codes for loci in the 268,345 parental map, in the format required for analysis using MAPQTL 6.0.

**Additional file 8.** 268,405 loc file. Plain text file containing the genotype codes for loci in the 268,405 parental map, in the format required for analysis using MAPQTL 6.0.

**Additional file 9.** All traits QTL pop. Plain text file containing the quantitative trait data of all individuals in the QTL population.

**Additional file 10.** 850,055 map file. Plain text file containing the map position (cM) of all loci on the 850,055 linkage map, in the format required for analysis using MAPQTL 6.0.

**Additional file 11.** 850,096 map file. Plain text file containing the map position (cM) of all loci on the 850,096 linkage map, in the format required for analysis using MAPQTL 6.0.

**Additional file 12.** 850,055 loc file. Plain text file containing the genotype codes for loci in the 850,055 parental map, in the format required for analysis using MAPQTL 6.0.

**Additional file 13.** 850,096 loc file. Plain text file containing the genotype codes for loci in the 850,096 parental map, in the format required for analysis using MAPQTL 6.0.

**Additional file 14.** All traits FWK pop. Plain text file containing the quantitative trait data of all individuals in the QTL population.

**Additional file 15: Table S4.** Comprehensive parental linkage maps constructed in the *Pinus radiata* QTL and FWK pedigrees: A) 268,345; B) 268,405; C) 850,055; D) 850,096.

**Additional file 16: Fig. S2.** Synteny and collinearity between linkage groups 8 and 10 amongst the *Pinus radiata* parental linkage maps in this study. Vertical bars represent linkage groups, horizontal lines within bars show the position of markers within each group, lines between groups indicate homologous markers at the contig level. Scale bars shows cM (Kosambi). Fig. S1A shows perfect synteny and high collinearity between these linkage groups in three parental linkage maps. **Fig. S2**B shows that linkage group 8 in parent 850,096 has markers from both linkage groups 8 and 10 in parents 268,345 and 268,405. However, all mapped markers in parent 850,096 matched only their homologous chromosome in parent 850,055 (Fig. S1C). The red box in fig. S1b highlights a region of ~ 10 cM, on linkage group 8 in parent 850,096, with a mixture of homologs from linkage groups 8 and 10 as well as very tightly clustered markers.

**Additional file 17: Table S5.** Matrix of Pearson’s phenotypic correlation coefficients between growth and wood property traits analysed in the *Pinus radiata* QTL population. Two tailed *P*-values *P < 0.05, **P < 0.01, ***P < 0.001, ****P < 0.0001.

**Additional file 18: Fig. S3.** Genomic predictive ability for diameter at breast height (DBH) and wood density (WD) as a function of training population size (A) and number of markers (B) used in random cross-validations of 81 *Pinus radiata* genotypes from the FWK population. Error bars correspond to standard deviations across 100 random cross-validations for each set of parameters. Analyses in (A) were based on all markers (*M* = 9353). The training population size in (B) was *N* = 40 (two-fold cross-validation) and subsets of markers were selected at random.

## Data Availability

The raw SNP genotype data used in all genomic analyses is publicly available in the Dryad repository, (https://datadryad.org/stash/dataset/doi:10.5061/dryad.03c7292). The phenotype data used in all analyses, and all input files for QTL analyses are included as Additional files (3 – 12).
