# Supplementary material for: High density linkage maps, genetic architecture, and genomic prediction of growth and wood properties in Pinus radiata
Source: BMC Genomics. 2022 Oct 28;23:731. doi: 10.1186/s12864-022-08950-6 (PMC9617409; doi:10.1186/s12864-022-08950-6)
Supplement: Supplementary file 1 — Additional file 1: Table S1. SNP filtering summary for the Pinus radiata QTL and FWK mapping populations. [file 12864_2022_8950_MOESM1_ESM.docx]

**Additional file 1: Table S1.** SNP filtering summary for the *Pinus radiata* QTL and FWK mapping populations

| **Filtering Step** | **Number of SNP** | | | |
| --- | --- | --- | --- | --- |
|  | **QTL population** | | **FWK population** | |
| Telfer et al. (9) preliminary filtering | 84,671 | | 75,413 | |
| Reproducibility of both parental genotypes > 50%, minimum 3 calls | 76,377 | | 62,938 | |
| Missing calls in offspring < 20 | 48,332 | | 36,042 | |
| Total test-cross markers | 22,676 | | 19,394 | |
| Test-cross markers by parent | 268345 | 268405 | 850055 | 850096 |
|  | 11,599 | 11,077 | 9,284 | 10,110 |
