# Supplementary material for: High density linkage maps, genetic architecture, and genomic prediction of growth and wood properties in Pinus radiata
Source: BMC Genomics. 2022 Oct 28;23:731. doi: 10.1186/s12864-022-08950-6 (PMC9617409; doi:10.1186/s12864-022-08950-6)
Supplement: Supplementary file 2 — Additional file 2: Table S2. SNP ranking criteria used for the Pinus radiata QTL and FWK mapping populations. [file 12864_2022_8950_MOESM2_ESM.docx]

**Additional file 2: Table S2.** SNP ranking criteria used for the *Pinus radiata* QTL and FWK mapping populations

| **Ranking Criteria** | **Class 1** | **Class 2** | **Class 3** |
| --- | --- | --- | --- |
| Parental reproducibility | 1 - 0.7 | 0.69 - 0.51 | 0.69 - 0.51 |
| PIC | 0.5 - 0.4 | 0.39 - 0.3 | 0.29 - 0.01 |
| Missing data | 0 - 9% | 10 - 14% | 15 - 20% |
