# Supplementary material for: High density linkage maps, genetic architecture, and genomic prediction of growth and wood properties in Pinus radiata
Source: BMC Genomics. 2022 Oct 28;23:731. doi: 10.1186/s12864-022-08950-6 (PMC9617409; doi:10.1186/s12864-022-08950-6)
Supplement: Supplementary file 3 — Additional file 3: Table S3. Descriptive statistics for the phenotypic traits measured in the QTL and FWK Pinus radiata populations in this study. [file 12864_2022_8950_MOESM3_ESM.docx]

| Population | Trait | Min | Max | Mean | StDev |
| --- | --- | --- | --- | --- | --- |
| QTL | Area (mm2) | 13769.6 | 105625 | 46945.18 | 15536.46 |
|  | Den (kg/m3) | 347.38 | 483.16 | 415.07 | 32.54 |
|  | Rad (µm) | 30.31 | 39.43 | 34.04 | 1.65 |
|  | Tan (µm) | 24.42 | 28.43 | 26.37 | 0.91 |
|  | Crs (µm/m) | 303.45 | 440.7 | 367.73 | 24.83 |
|  | Wall (µm) | 1.88 | 2.55 | 2.2 | 0.15 |
|  | Sur (m2/kg) | 290.74 | 384.77 | 336.72 | 21.28 |
|  | MFA (degrees) | 26.57 | 39.01 | 32.23 | 2.69 |
|  | MOE (GPa) | 2.68 | 7.25 | 4.83 | 0.96 |
|  | CORE_A (kg/m3) | 266 | 357 | 307.31 | 29.28 |
|  | CORE_B (kg/m3) | 266 | 359 | 312.07 | 29.24 |
|  | JWD (kg/m3) | 272 | 349.5 | 309.69 | 28.69 |
|  | DBH (mm) | 40 | 121 | 86.92 | 20.63 |
| FWK | LW1-10 (%) | 1.5 | 23 | 7.59 | 3.98 |
|  | WD (kg/m3) | 328.99 | 407.09 | 364.99 | 19.34 |
|  | DBH (mm) | 157 | 461 | 327.98 | 64.22 |

**Additional file 3: Table S3.** Descriptive statistics for the phenotypic traits measured in the QTL and FWK *Pinus radiata* populations in this study.
