# Supplementary figures and images for: High density linkage maps, genetic architecture, and genomic prediction of growth and wood properties in Pinus radiata
Source: BMC Genomics. 2022 Oct 28;23:731. doi: 10.1186/s12864-022-08950-6 (PMC9617409; doi:10.1186/s12864-022-08950-6)

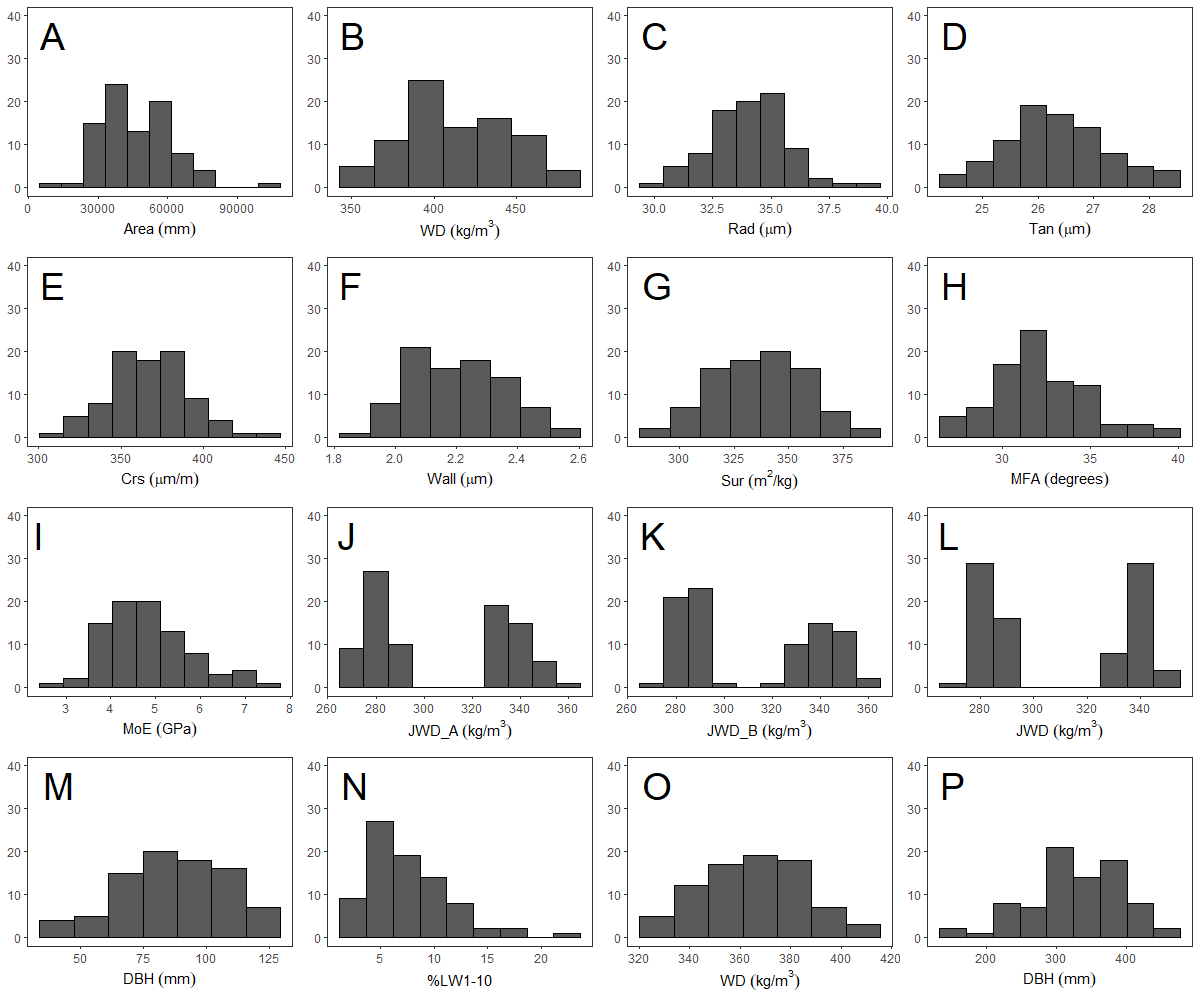

Supplement: Supplementary file 4 — Additional file 4: Fig. S1. Frequency distributions for the phenotypic traits measured in the QTL and FWK Pinus radiata populations in this study. QTL population: (A) Ring area (mm2); (B) Density (kg/m3) Silviscan; (C) Radial cell diameter (μm); (D) Tangential cell diameter (μm); (E) Fibre coarseness (μm/m); (F) Cell wall thickness (μm); (G) Specific surface area (m2/kg); (H) Microfibril angle (degrees); (I) Modulus of elasticity (GPa); (J) Density prediction for first 5 mm core (maximum moisture content method) (kg/m3); (K) Density prediction for second 5 mm core (maximum moisture content method) (kg/m3); (L) Average of density predictions for two cores above; (M) Diameter at breast height (mm). FWK population: (N) Area weighted percent late wood ages 1–10 (%); (O) Wood density (kg/m3) (maximum moisture content method); (P) Diameter at breast height (mm). [file 12864_2022_8950_MOESM4_ESM.docx]

## Slide 1
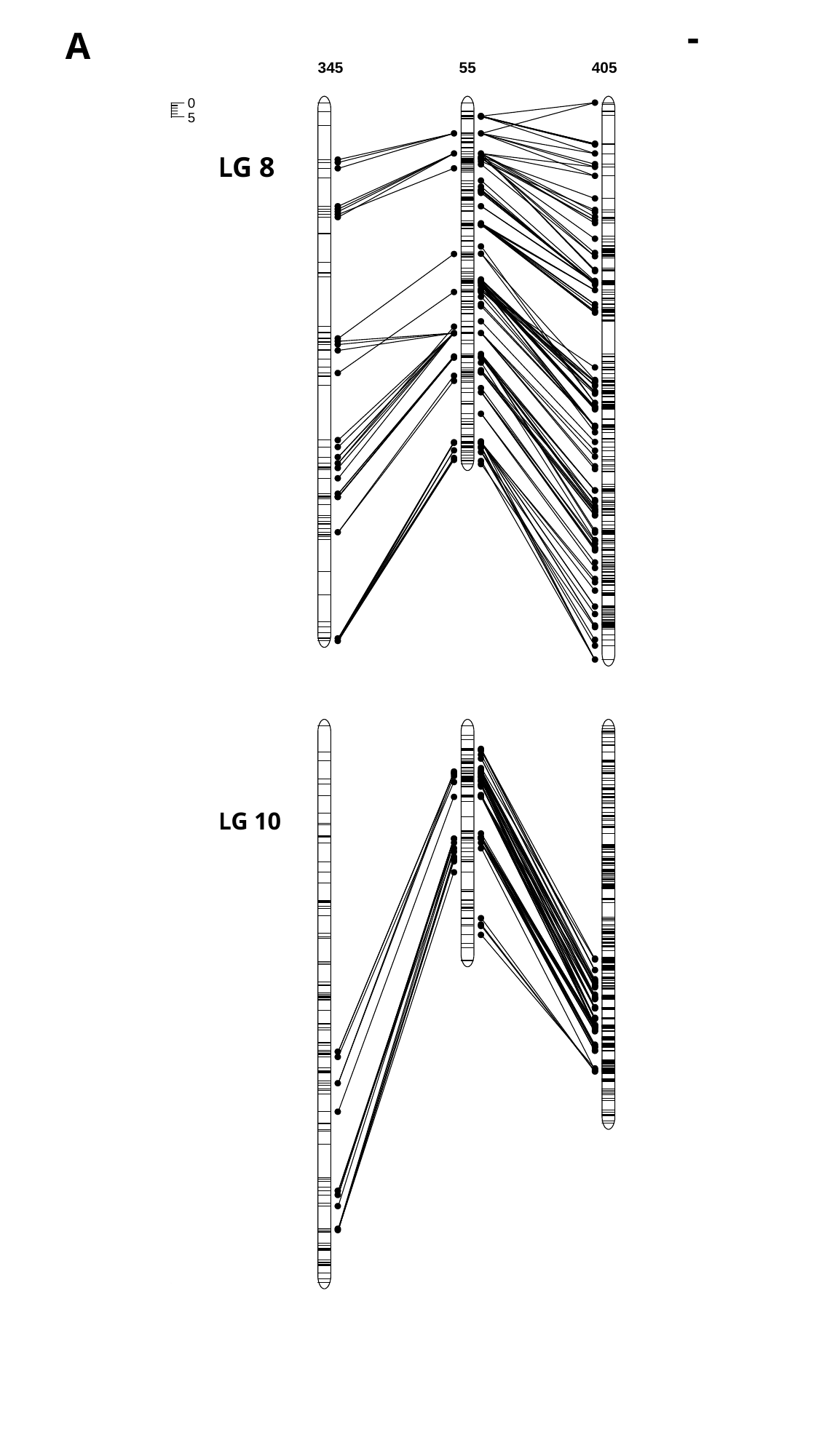

A
LG 8
LG 10

## Slide 2
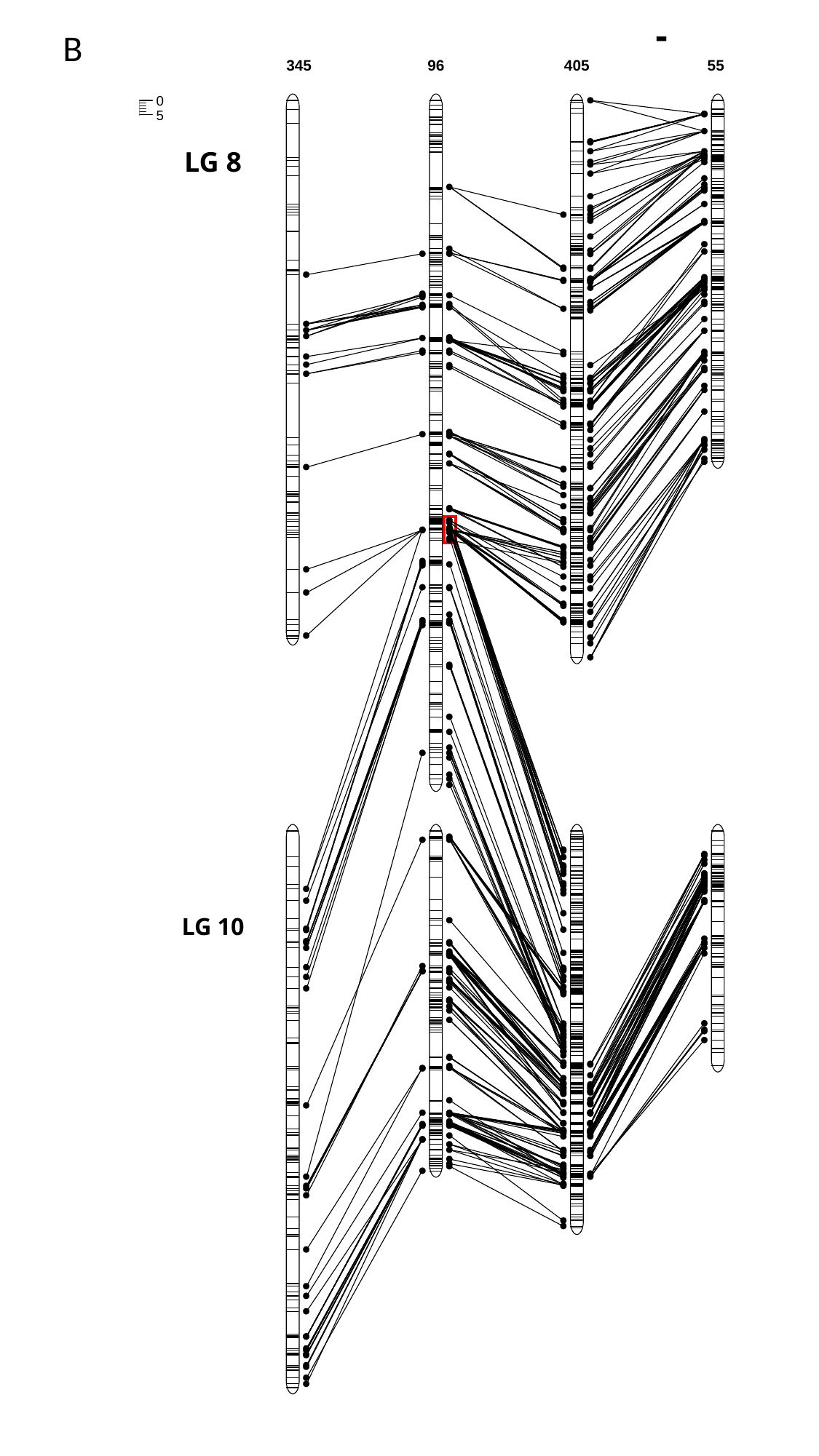

B
LG 8
LG 10

## Slide 3
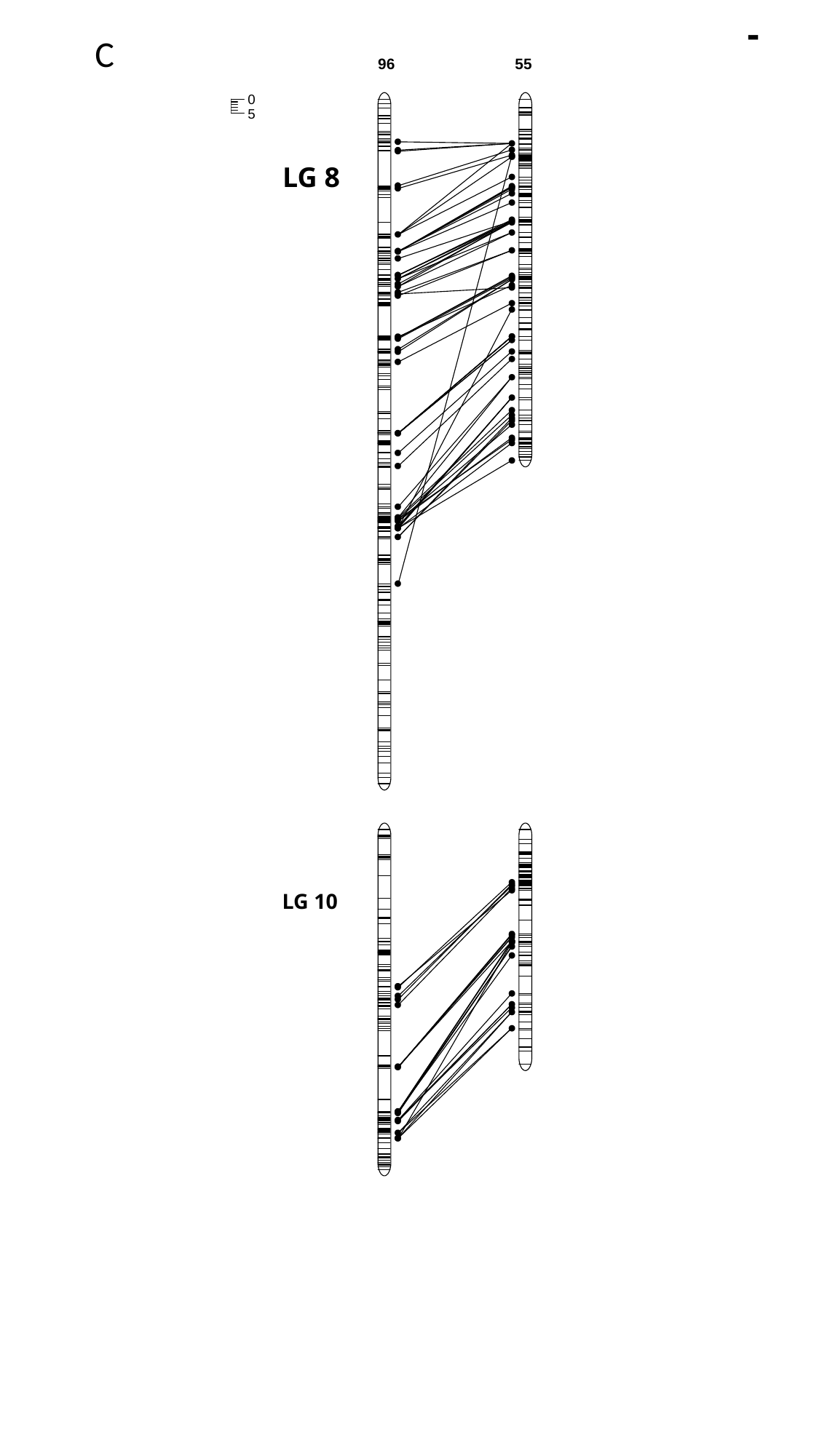

C
LG 8
LG 10

Supplement: Supplementary file 16 — Additional file 16: Fig. S2. Synteny and collinearity between linkage groups 8 and 10 amongst the Pinus radiata parental linkage maps in this study. Vertical bars represent linkage groups, horizontal lines within bars show the position of markers within each group, lines between groups indicate homologous markers at the contig level. Scale bars shows cM (Kosambi). Fig. S1A shows perfect synteny and high collinearity between these linkage groups in three parental linkage maps. Fig. S2B shows that linkage group 8 in parent 850,096 has markers from both linkage groups 8 and 10 in parents 268,345 and 268,405. However, all mapped markers in parent 850,096 matched only their homologous chromosome in parent 850,055 (Fig. S1C). The red box in fig. S1b highlights a region of ~ 10 cM, on linkage group 8 in parent 850,096, with a mixture of homologs from linkage groups 8 and 10 as well as very tightly clustered markers. [file 12864_2022_8950_MOESM16_ESM.pptx]

## Slide 1
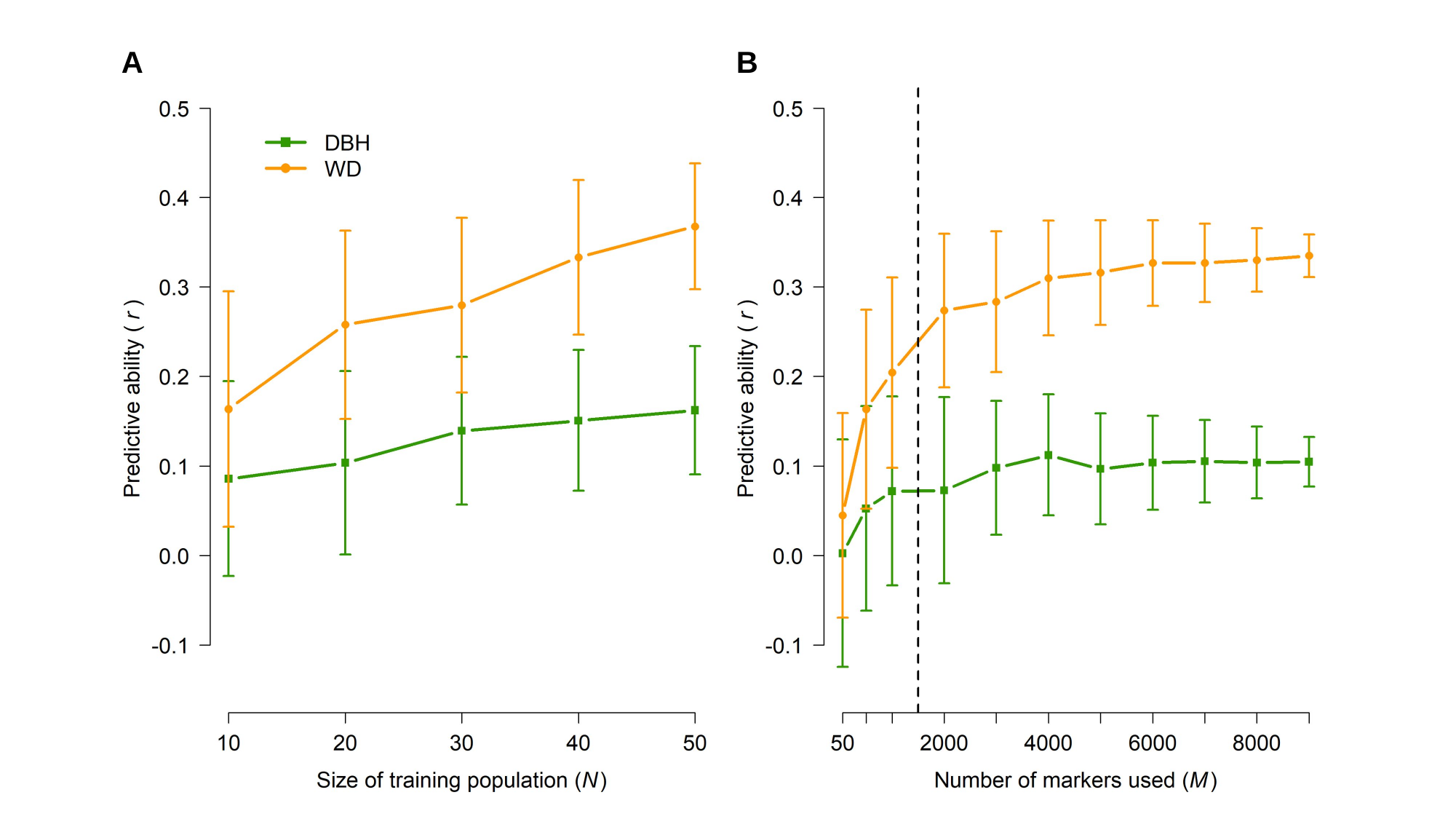

A
B

Supplement: Supplementary file 18 — Additional file 18: Fig. S3. Genomic predictive ability for diameter at breast height (DBH) and wood density (WD) as a function of training population size (A) and number of markers (B) used in random cross-validations of 81 Pinus radiata genotypes from the FWK population. Error bars correspond to standard deviations across 100 random cross-validations for each set of parameters. Analyses in (A) were based on all markers (M = 9353). The training population size in (B) was N = 40 (two-fold cross-validation) and subsets of markers were selected at random. [file 12864_2022_8950_MOESM18_ESM.ppt]
