# Supplementary material for: High density linkage maps, genetic architecture, and genomic prediction of growth and wood properties in Pinus radiata
Source: BMC Genomics. 2022 Oct 28;23:731. doi: 10.1186/s12864-022-08950-6 (PMC9617409; doi:10.1186/s12864-022-08950-6)
Supplement: Supplementary file 17 — Additional file 17: Table S5. Matrix of Pearson’s phenotypic correlation coefficients between growth and wood property traits analysed in the Pinus radiata QTL population. Two tailed P-values *P < 0.05, **P < 0.01, ***P < 0.001, ****P < 0.0001. [file 12864_2022_8950_MOESM17_ESM.docx]

**Additional file 17: Table S5**. Matrix of Pearson’s phenotypic correlation coefficients between growth and wood property traits analysed in the *Pinus radiata* QTL population.

|  | Area | WD | Rad | Tan | Crs | Wall | Sur | MFA | MOE | JWD_A | JWD_B | JWD |
| --- | --- | --- | --- | --- | --- | --- | --- | --- | --- | --- | --- | --- |
| WD | -0.27* |  |  |  |  |  |  |  |  |  |  |  |
| Rad | 0.22 | -0.51**** |  |  |  |  |  |  |  |  |  |  |
| Tan | 0.35** | -0.63**** | 0.48**** |  |  |  |  |  |  |  |  |  |
| Crs | 0.03 | 0.56**** | 0.34** | 0.09 |  |  |  |  |  |  |  |  |
| Wall | -0.16 | 0.93**** | -0.21 | -0.37** | 0.82**** |  |  |  |  |  |  |  |
| Sur | 0.14 | -0.92**** | 0.21 | 0.35** | -0.83**** | -0.99**** |  |  |  |  |  |  |
| MFA | 0.28* | 0.06 | -0.14 | -0.25* | -0.14 | -0.03 | 0.00 |  |  |  |  |  |
| MOE | -0.39*** | 0.40*** | -0.20 | -0.01 | 0.34** | 0.44**** | -0.40*** | -0.82**** |  |  |  |  |
| JWD_A | -0.37** | 0.68**** | -0.37** | -0.58**** | 0.27* | 0.58**** | -0.58**** | 0.05 | 0.22 |  |  |  |
| JWD_B | -0.33** | 0.67**** | -0.35** | -0.52**** | 0.32** | 0.60**** | -0.60**** | 0.07 | 0.22 | 0.92**** |  |  |
| JWD | -0.36** | 0.69**** | -0.37** | -0.56**** | 0.30** | 0.60**** | -0.60**** | 0.06 | 0.22 | 0.98**** | 0.98**** |  |
| DBH | 0.54**** | -0.14 | -0.05 | 0.33 | -0.02 | -0.09 | 0.09 | 0.08 | -0.05 | -0.28* | -0.22 | -0.25* |

Two tailed P-values *P<0.05, **P<0.01, ***P<0.001, ****P<0.0001
